# Accuracy of echocardiographic indices for serial monitoring of right ventricular systolic function in patients with precapillary pulmonary hypertension

**DOI:** 10.1371/journal.pone.0187806

**Published:** 2017-11-09

**Authors:** Takahiro Sato, Ichizo Tsujino, Hiroshi Ohira, Noriko Oyama-Manabe, Yoichi M. Ito, Chisa Takashina, Taku Watanabe, Masaharu Nishimura

**Affiliations:** 1 First Department of Medicine, Hokkaido University Hospital, Sapporo, Japan; 2 Department of Diagnostic and Interventional Radiology, Hokkaido University Hospital, Sapporo, Japan; 3 Department of Biostatistics, Hokkaido University Graduate School of Medicine, Sapporo, Japan; Temple University, UNITED STATES

## Abstract

**Background:**

Serial assessment of right ventricular ejection fraction (RVEF) predicts the clinical outcome of patients with pulmonary hypertension (PH). Cardiac magnetic resonance imaging (CMRI) enables RVEF monitoring, but its applicability is limited in clinical practice. This study aimed to examine the correlation between changes in CMRI-derived RVEF with those in echocardiographic indices in patients with precapillary PH.

**Methods:**

CMRI and echocardiographic indices of RV systolic function were evaluated at baseline and follow-up in 54 consecutive patients with precapillary PH (pulmonary arterial hypertension (PAH), n = 23; non-PAH, n = 31). During follow-up, medical treatment was optimized according to the guidelines for PH. Using CMRI-derived RVEF as the gold standard, we examined the accuracy of five echocardiographic indices by correlation analysis and receiver operating characteristic (ROC) analysis and by calculating sensitivity, specificity, and positive and negative predictive values.

**Results:**

After an average period of 9.5 months, CMRI-derived RVEF improved from 30.2% ± 10.6% at baseline to 41.4% ± 11.3% at follow-up. These changes significantly correlated with those in the five echocardiographic indices, i.e., %RV fractional shortening (r = 0.27), %RV area change (r = 0.46), tricuspid annular plane systolic excursion (TAPSE) (r = 0.84), RV myocardial performance index (RVMPI) (r = −0.72), and systolic lateral tricuspid annular motion velocity (TVlat) (r = 0.66). Of these indices, %RV area change, TAPSE, and TVlat significantly correlated with those of CMRI-derived RVEF in both PAH and non-PAH subgroups. ROC analysis showed that improvement in echocardiographic indices predicted a pre-specified improvement in CMRI-derived RVEF (>2.9%), with TAPSE and TVlat showing better accuracy over the other three indices.

**Conclusions:**

Echocardiographic indices modestly correlate with the changes in CMRI-derived RVEF in precapillary PH patients. Comparison among the five echocardiographic indices revealed that TAPSE and TVlat provide better accuracy than %RV fractional shortening, %RV area change, and RVMPI.

## Introduction

In pulmonary hypertension (PH), chronic elevation of pulmonary arterial pressure (PAP) causes right ventricular (RV) dysfunction with its related symptoms and signs, as well as premature death.[1–3] Therefore, precise evaluation of RV morphology and function is crucial in the management of PH. The complex geometry of the RV chamber has hampered its accurate assessment, but the recent progress in cardiac magnetic resonance imaging (CMRI) has enabled precise calculation of RV ejection fraction (RVEF) in patients with PH.[4, 5] Moreover, the latest guidelines for PH introduce CMRI as an accurate modality for the evaluation of RV morphology and function [6]; recommended parameters of this guideline include RV volume, stroke volume, and ejection fraction. The guidelines also address the importance of serial RV assessment on the basis of recent CMRI reports that showed its utility in the long-term management of PAH by identifying RV failure prior to the development of clinical features.[6–8] However, the clinical application of CMRI for RV evaluation is still limited because of the need for a dedicated facility, analysis application, expertise, and contraindications in patients with implanted devices.

Echocardiography is a noninvasive and accessible modality suitable for repeated evaluation of RV morphology and function. Dedicated echocardiographic guidelines for RV assessment recommend the use of %RV area change, tricuspid annular plane systolic excursion (TAPSE), and/or Doppler parameters for evaluating RV systolic function.[9] More recent publications on PH have shown that echocardiographic RV parameters, such as %RV area change and TAPSE, correlated well with CMRI-derived RVEF.[10–13] The clinical relevance of evaluating RV function by echocardiography has also been reported in various types of PH, including pulmonary arterial hypertension (PAH),[6, 14] left heart disease-associated PH,[15] lung disease-associated PH,[16] and chronic thromboembolic pulmonary hypertension (CTEPH).[17] However, echocardiography is known to encounter a challenge in obtaining clear images, particularly in PH patients with a severely dilated right heart or comorbid advanced lung disease.[18] In addition, most previous studies were cross-sectional in nature, and only a few have examined the accuracy of serial monitoring of echocardiographic indices of RV systolic function[19]. With the remarkable progress in the treatments of PH, there is a need for the development of simple, noninvasive, and reproducible methods to assess RV function in clinical practice.

In this retrospective study, we sought to examine the accuracy of echocardiographic indices for monitoring RV systolic function in medically treated patients with precapillary PH using CMRI-derived RVEF as a gold standard.

## Materials and methods

### Patient selection

We retrospectively analyzed consecutive patients with precapillary PH diagnosed by right heart catheterization (RHC) between April 2010 and February 2013. Inclusion criteria were mean PAP of ≥25 mmHg and pulmonary artery wedge pressure of ≤15 mmHg.[20] Exclusion criteria were an inability to obtain or analyze the CMRI or echocardiography images; concomitant left heart disease, including coronary, valvular, and myocardial diseases, and the presence of atrial fibrillation/flutter.

This study was approved by the ethics committee of the Hokkaido University Hospital, and written informed consent was obtained from all patients.

### Echocardiography

**Fig 1:**
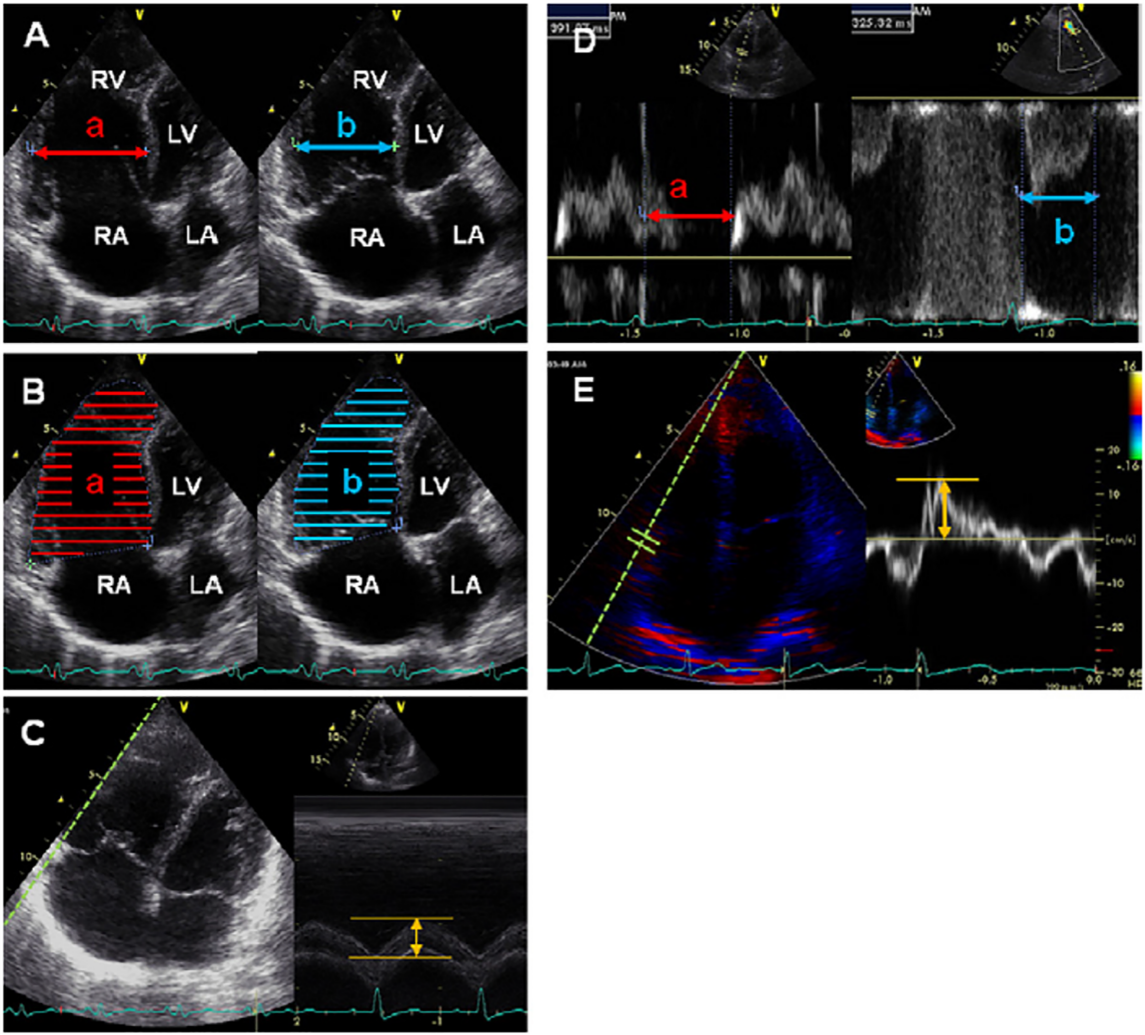
Representative images of echocardiographic assessment of right ventricular systolic function. (A) %RVFS [(a–b)/a × 100%]; (B) percentage RV area change [(a–b)/a × 100%]; (C) TAPSE; (D) right ventricular MPI [(a–b)/b]; and (E) TVlat. See text for further information on how these indices were obtained or calculated. LA, left atrium; LV, left ventricle; RA, right atrium; RV, right ventricle. Reprinted from J Am Soc Echocardiogr. 2012 Mar;25(3):280–6, Takahiro Sato et al., Validation study on the accuracy of echocardiographic measurements of right ventricular systolic function in pulmonary hypertension,[7] with permission from Elsevier.

Echocardiograms were obtained using Vivid q and Vivid S6 (GE Healthcare, Milwaukee, WI, USA) and images were analyzed offline after recording. Five echocardiographic parameters were obtained as previously reported;[10, 21–24] the representative images for all indices are shown in Fig 1. RV fractional shortening (FS) was measured in the two-dimensional, apical four-chamber view by calculating regional changes in the distance between the interventricular septum to the RV free wall at the mid-RV level between end-diastole and end-systole. Using apical four chamber views, %RV area change was calculated by dividing the difference in RV area between end-diastolic and end-systolic phases by the end-diastolic RV area. TAPSE was measured using M-mode apical four-chamber images, wherein the cursor was oriented to the junction of the tricuspid valve plane and the RV free wall; total displacement of the tricuspid annulus from end-diastole to end-systole was measured.[21, 22] The RV myocardial performance index (RV MPI) was calculated by the following equation:

RVMPI=(a−b)/b

where (a) represented the interval between cessation and onset of tricuspid flow velocity and (b) represented RV outflow velocity time.[23] Systolic lateral tricuspid annular motion velocity (TVlat) was measured in the apical four-chamber view on pulsed-wave tissue Doppler imaging (TDI) mode while the ultrasound cursor was carefully placed parallel to the direction of the tricuspid annular motion.[24]

Image acquisition and analysis were performed by an experienced cardiologist (T.S.) who was blinded to the CMRI measurements.

### Cardiac magnetic resonance imaging

CMRI studies were performed on a 1.5-Tesla Philips Achieva MRI system (Philips Medical Systems, Best, The Netherlands) using master gradients (maximum gradient amplitude 33 mT/m and maximum slew rate 100 mT/m/ms), as reported in our previous study.[10] With subjects in the supine position, imaging was performed using a five-element, cardiac-phased array coil with breath holding in expiration and a vector cardiography method for electrocardiogram gating. Localizing scans were followed by breath hold cine imaging in the axial plane. A total of 12 axial slices were acquired using a steady-state, free-precession pulse sequence (time to repeat [TR] = 2.8 ms, time to echo [TE] = 1.4 ms, flip angle = 60°, acquisition matrix = 192 × 256, field of view = 380 mm, slice thickness = 10 mm, 0 mm interslice gap, and 20 phases/cardiac cycle).

Offline image analysis of the RV was performed using commercially available software (Extended MR Work Space: v 2.6.3; Philips Medical Systems, Amsterdam, The Netherlands), according to the methods reported by Alfakih.[25] Briefly, in the electrocardiogram-gated axial data sets, the endocardial contours of the RV were traced manually at the first phase and at two phases that had the smallest volumes in the middle of the cardiac cycle. The workstation selected the first phase of each slice as end-diastole and the smaller of the two middle phases as end-systole. The RV contour was traced up to the tricuspid valve and was closed by a straight line across the tricuspid valve ring. The most apical slice was defined as the last slice that contained blood volume. RV end-diastolic volume (EDV) and end-systolic volume (ESV) were computed. Stroke volume (SV) was calculated as EDV − ESV, whereas ejection fraction (EF) was calculated as SV/EDV × 100%. CMRI analysis was performed by an experienced radiologist (N.M.) who was blinded to the results of echocardiographic evaluations.

### Baseline and follow-up assessments

Within a week of being clinically stable, patients who met the inclusion criteria were assessed at baseline using World Health Organization (WHO) functional classification, plasma brain natriuretic peptide (BNP) concentration, echocardiography, and CMRI. During this period, all patients were hospitalized and were studied only when their PH-related symptoms (e.g., shortness of breath or edema) and vital signs (e.g., blood pressure, heart rate, and oxygen saturation) were stable.

Patients with PAH were treated according to the goal-oriented strategy introduced in the PH guidelines published in 2009.[20, 26] The treatment was optimized after baseline assessment of PH if needed. Patients with PH subtypes other than PAH were managed according to the same guidelines, but were allowed to receive drugs approved for PAH by the Japanese healthcare system when the doctor considered it clinically beneficial. A second assessment was conducted after modification of the treatment for PH or as part of a regular check-up. The examinations on the second assessment were the same as those for at baseline and included RHC, CMRI, and echocardiography. The patients underwent these assessments within a week of hospitalization and in a stable condition without treatment modification.

### Statistical analysis

We first analyzed the correlation between CMRI-derived RVEF and the five echocardiographic measurements at baseline and follow-up then examined the correlation between changes in both measurements.

Receiver operating characteristic (ROC) analysis was performed to examine the ability of the echocardiographic indices to detect significant improvement in CMRI-derived RVEF. The cut-off level for significant improvement of CMRI-derived RVEF was determined as 2.9% based on the standard deviation (SD) of the interobserver variation reported by a previous study that used the same methodology of calculating RVEF.[25]

Using CMRI-derived RVEF as a gold standard, we calculated the sensitivity, specificity, positive predictive value (PPV), and negative predictive value (NPV) of the five echocardiographic indices of RV contraction. Similar to the ROC analysis, an increase of > 2.9% was used as the cut-off value for significant improvement in CMRI-derived RVEF. To obtain the cut-off values for the echocardiographic indices, we used our previously reported SDs of interobserver variations of each index,[10] as follows: 3.0% for %RV FS, 7.9% for %RV area change, 1.1 mm for TAPSE, 0.07 for RVMPI, and 0.25 cm/s for TVlat.

Correlation and ROC analyses and the calculations of sensitivity, specificity, PPV, and NPV were conducted in all precapillary PH patients and in the PAH and non-PAH subgroups, considering the possible differences in the demographics and the PH-related clinical data between the PAH and non-PAH subgroups.

JMP^®^ Version 12 (SAS Institute Inc., Cary, NC) was used for statistical analyses and a p value of < 0.05 was considered statistically significant.

## Results

In total, 82 Japanese patients were diagnosed with precapillary PH by RHC during the study period. Among these, 54 patients (66%) underwent baseline and follow-up RHC, CMRI, and echocardiography. %RV area changes at baseline and follow-up were not calculated in two patients (one PAH, one non-PAH) because of poor image quality, whereas the other four echocardiographic indices were evaluated in all 54 patients

The baseline clinical characteristics of the 54 patients are shown in Table 1. Of these, 23 (43%) were diagnosed with PAH and the remaining 31 patients (57%) were diagnosed as non-PAH PH. The latter group included patients with respiratory disease-associated PH (n = 9), CTEPH (n = 17), and PH due to other causes (n = 5).

**Table 1:** Baseline patient characteristics.

Number of patients			54
Gender (male/female)			13/41
Age (years)			56 ± 16; range, 28–86
Subtype of pulmonary hypertension			
	Pulmonary arterial hypertension		23 (43%)
		Idiopathic/heritable	9
		Connective tissue disease-associated	10
		Other PAH/PVOD/PCH	4
	Respiratory disease-associated		9 (17%)
	CTEPH		17 (31%)
	Others type		5 (9%)

PAH, pulmonary arterial hypertension; PVOD, pulmonary venoocclusive disease; PCH, pulmonary capillary hemangiomatosis; CTEPH, chronic thromboembolic pulmonary hypertension

As shown in Table 2, the comparison between PAH and non-PAH groups showed that the female/male ratio was higher and that the age was younger in the PAH group than in the non-PAH group. PAH-approved drugs were used more in the PAH group (16/23, 69.6%) than in the non-PAH group (11/31, 35.5%) (p = 0.013). In addition, baseline RHC and CMRI data showed that CI and RVEF were significantly higher and that PAWP was lower in the PAH group than in the non-PAH group, whereas there were no significant differences in the other indices between the two groups.

**Table 2:** Demographics and baseline right heart catheterization and cardiac magnetic resonance imaging data of PAH and non-PAH patients.

	PAH group	Non-PAH group	p-value
Number of subjects	23	31	
Female/Male	21/2	20/11	0.028
Age (years)	49.8 ± 3.1	61.3 ± 2.7	0.007
Use of PAH drugs, Yes/No	16/7	11/20	0.013
Pulmonary hemodynamics			
Pulmonary arterial wedge pressure (mmHg)	8 ± 3	6 ± 3	0.016
Mean pulmonary arterial pressure (mmHg)	41 ± 11	38 ± 12	0.353
Cardiac index (L/min/m^2^)	2.9 ± 0.8	2.4 ± 0.6	0.009
Pulmonary vascular resistance (dyne·s·cm^−5^)	638 ± 305	772 ± 381	0.171
CMRI indices			
RV end-diastolic volume index (ml/m^2^)	109.2 ± 38.3	112.0 ± 51.4	0.830
RV end-systolic volume index (ml/m^2^)	68.4 ± 29.9	76.4 ± 44.9	0.462
RVEF (%)	39.6 ± 6.8	33.8 ± 12.3	0.045

PAH, pulmonary arterial hypertension; CMRI, cardiac magnetic resonance imaging; RV, right ventricular; RVEF, right ventricular ejection fraction

Table 3 shows the WHO functional class (WHO-FC), plasma BNP level, 6-minute walk distance, treatment regimens, and measurements of RHC, CMRI, and echocardiography at baseline and follow-up. All patients underwent follow-up hemodynamic assessment at an average period of 9.5 months (range, 3–31 months) after baseline RHC. During follow-up of the 27 patients treated with PAH-approved drugs at baseline, 14 patients additionally received new PAH-approved drug(s), one received a dose increase, and two discontinued the PAH-approved drug(s). In the 27 PAH patients naïve to any PAH drug at baseline, PAH-approved drugs were newly initiated in 22 patients during follow-up. As a result, the number of patients on PAH-approved drugs increased from 50% (27/54) at baseline to 87% (47/54) on follow-up. During follow-up, mean PAP decreased, CI increased, and pulmonary vascular resistance declined with a statistical significance (p < 0.001). CMRI-derived RVEF also improved from 36.2% ± 10.6% to 41.4% ± 11.3% (p < 0.001) during follow-up. Among the echocardiographic indices, TAPSE, RVMPI, and TVlat significantly improved, whereas %RV area change and RVFS did not.

**Table 3:** Clinical parameters and treatment regimens for pulmonary hypertension at baseline and follow-up.

		Baseline	Follow-up	p-value
Number of subjects		54	54	
WHO-functional class (I/II/III/IV)		0/18/25/11	1/25/22/6	0.011
BNP concentration (pg/mL)		302 ± 479	145 ± 221	0.009
6-minute walk distance (m)		343 ± 147	401 ± 128	0.016
Number of PAH drugs given				
	0	27	7	
	1 (monotherapy)	13	18	
	2 (dual combination)	11	19	
	3 (triple combination)	3	10	
	Either of 1, 2, or 3	27 (50.0%)	47 (87.0%)	
Endothelin receptor antagonist		14	28	
Phosphodiesterase 5 inhibitor		11	38	
Oral prostacyclin		17	20	
Intravenous prostacyclin		2	2	
Domiciliary oxygen treatment		18 (33.3%)	30 (55.6)	
Pulmonary hemodynamics				
	Heart rate (/min)	74 ± 13	74 ± 13	0.909
	Systolic blood pressure (mmHg)	116 ± 17	113 ± 18	0.087
	Diastolic blood pressure (mmHg)	67 ± 13	64 ± 13	0.049
	Pulmonary arterial wedge pressure (mmHg)	7 ± 3	8 ± 3	0.164
	Mean pulmonary arterial pressure (mmHg)	39 ± 12	35 ± 12	0.006
	Mean right atrial pressure (mmHg)	6 ± 4	5 ± 3	0.184
	Cardiac index (L/min/m^2^)	2.6 ± 0.7	2.8 ± 0.8	0.007
	Pulmonary vascular resistance (dyne·s·cm^−5^)	714 ± 354	552 ± 282	< 0.001
CMRI indices				
	RV end-diastolic volume index (ml/m^2^)	170.4 ± 67.4	155.5 ± 58.3	0.014
	RV end-systolic volume index (ml/m^2^)	113.2 ± 57.3	94.6 ± 46.1	< 0.001
	RVEF (%)	36.2 ± 10.6	41.4 ± 11.3	< 0.001
Echocardiographic indices				
	%RV fractional shortening (%)	18.1 ± 9.7	17.6 ± 8.0	0.651
	%RV area change (%)*	28.6 ± 11.5	31.4 ± 11.7	0.053
	TAPSE (mm)	17.7 ± 4.9	19.8 ± 4.8	0.002
	RV MPI	0.55 ± 0.29	0.47 ± 0.21	0.028
	TVlat (cm/s)	9.0 ± 2.5	10.0 ± 2.8	0.002

*%RV area changes at baseline and follow-up were not calculated in two patients (one PAH, one non-PAH) because of poor image quality, whereas the other four echocardiographic indices were evaluated in all 54 patients.

WHO, World Health Organization; BNP, brain natriuretic peptide; CMRI, cardiac magnetic resonance imaging; RV, right ventricular; RVEF, right ventricular ejection fraction; TAPSE, tricuspid annular plane systolic excursion; RV MPI, right ventricular myocardial performance index; TVlat, systolic lateral tricuspid annular motion velocity

### Correlation of the five echocardiographic parameters with CMRI-derived RVEF

As shown in Table 4, all five echocardiographic indices significantly correlated with CMRI-derived RVEF at baseline and follow-up. In the subgroup analysis of the PAH and non-PAH subgroups, similar significant correlations were observed, except for follow-up %RV area change, baseline and follow-up RVMPI of the PAH group, and follow-up RVFS of the non-PAH group.

**Table 4:** Associations of the five echocardiographic parameters with cardiac magnetic resonance imaging-derived right ventricular ejection fraction (N = 54).

	Baseline		Follow-up		Δ (Follow-up—Baseline)	
	r	p	r	p	r	p
RVFS	0.48	<0.001	0.54	<0.001	0.27	0.047
%RV area change*	0.59	<0.001	0.54	<0.001	0.46	<0.001
TAPSE	0.87	<0.001	0.88	<0.001	0.84	<0.001
RV MPI	−0.55	<0.001	−0.53	<0.001	0.72	<0.001
TVlat	0.62	<0.001	0.59	<0.001	0.66	<0.001
PAH patients (n = 23)						
RVFS	0.56	0.006	0.53	0.01	0.23	0.264
%RV area change*	0.57	0.006	0.39	0.071	0.38	0.06
TAPSE	0.65	<0.001	0.83	<0.001	0.78	<0.001
RV MPI	−0.34	0.117	−0.34	0.115	0.62	<0.001
TVlat	0.5	0.016	0.64	0.001	0.54	<0.005
Non-PAH patients (n = 31)						
RVFS	0.5	0.006	0.39	0.07	0.28	0.155
%RV area change	0.58	<0.001	0.57	0.001	0.51	0.006
TAPSE	0.92	<0.001	0.9	<0.001	0.91	<0.001
RV MPI	−0.67	<0.001	−0.63	<0.001	0.8	<0.001
TVlat	0.69	<0.001	0.55	0.002	0.76	<0.001

*%RV area changes at baseline and follow-up were not calculated in two patients (one PAH, one non-PAH) because of poor image quality, whereas the other four echocardiographic indices were evaluated in all 54 patients.

RVFS, right ventricular fractional shortening; RV, right ventricular; TAPSE, tricuspid annular plane systolic excursion; RV MPI, right ventricular myocardial performance index; TVlat, systolic lateral tricuspid annular motion velocity

**Fig 2:**
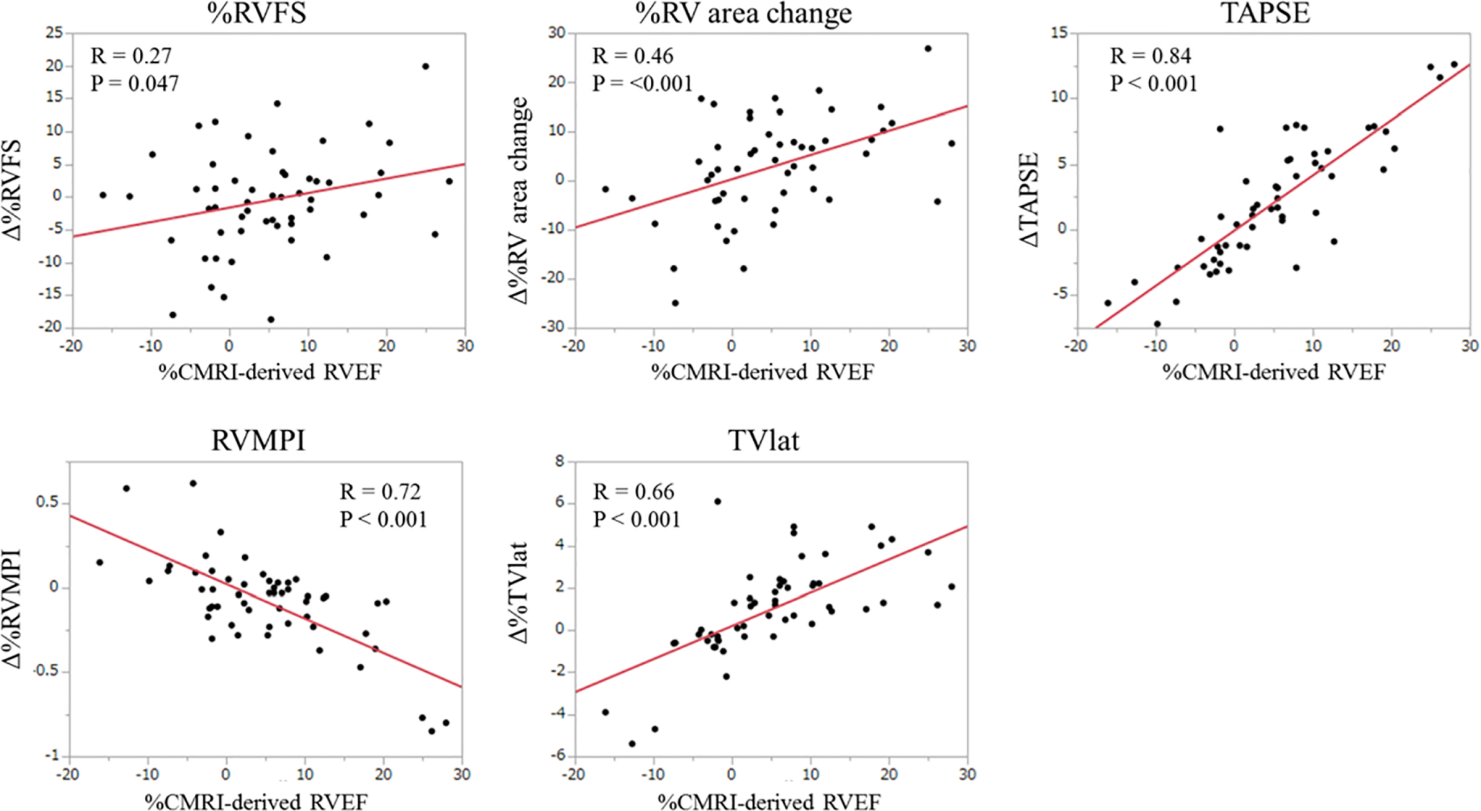
Correlation between the changes in CMRI-derived RVEF and echocardiographic parameters. There were significant correlations between the changes in CMRI-derived RVEF and those in the five echocardiographic indices. %RV area changes at baseline and follow-up were not calculated in two patients (one PAH, one non-PAH) because of poor image quality, whereas the other four echocardiographic indices were evaluated in all 54 patients. CMRI, cardiac magnetic resonance imaging; RV, right ventricular; RVEF, right ventricular ejection fraction; RVFS, right ventricular fractional shortening; TAPSE, tricuspid annular plane systolic excursion; RV MPI, right ventricular myocardial performance index; TVlat, systolic lateral tricuspid annular motion velocity.

The changes in all five echocardiographic indices were significantly correlated with the changes in CMRI-derived RVEF (Fig 2). Among the five echocardiographic indices, the changes in TAPSE exhibited the highest correlation coefficient with those in CMRI-derived RVEF (r = 0.84). In the subgroup analysis on the PAH group, the changes in RVMPI, TAPSE, and TVlat were significantly correlated with those in CMRI-derived RVEF, but %RV FS and %RV area change were not. In the non-PAH group, the changes in %RV area change, TAPSE, RV MPI, and TVlat were significantly correlated with those in CMRI-derived RVEF, but %RV FS was not.

### Ability of echocardiographic parameters to detect significant improvement in CMRI-derived RVEF

**Fig 3:**
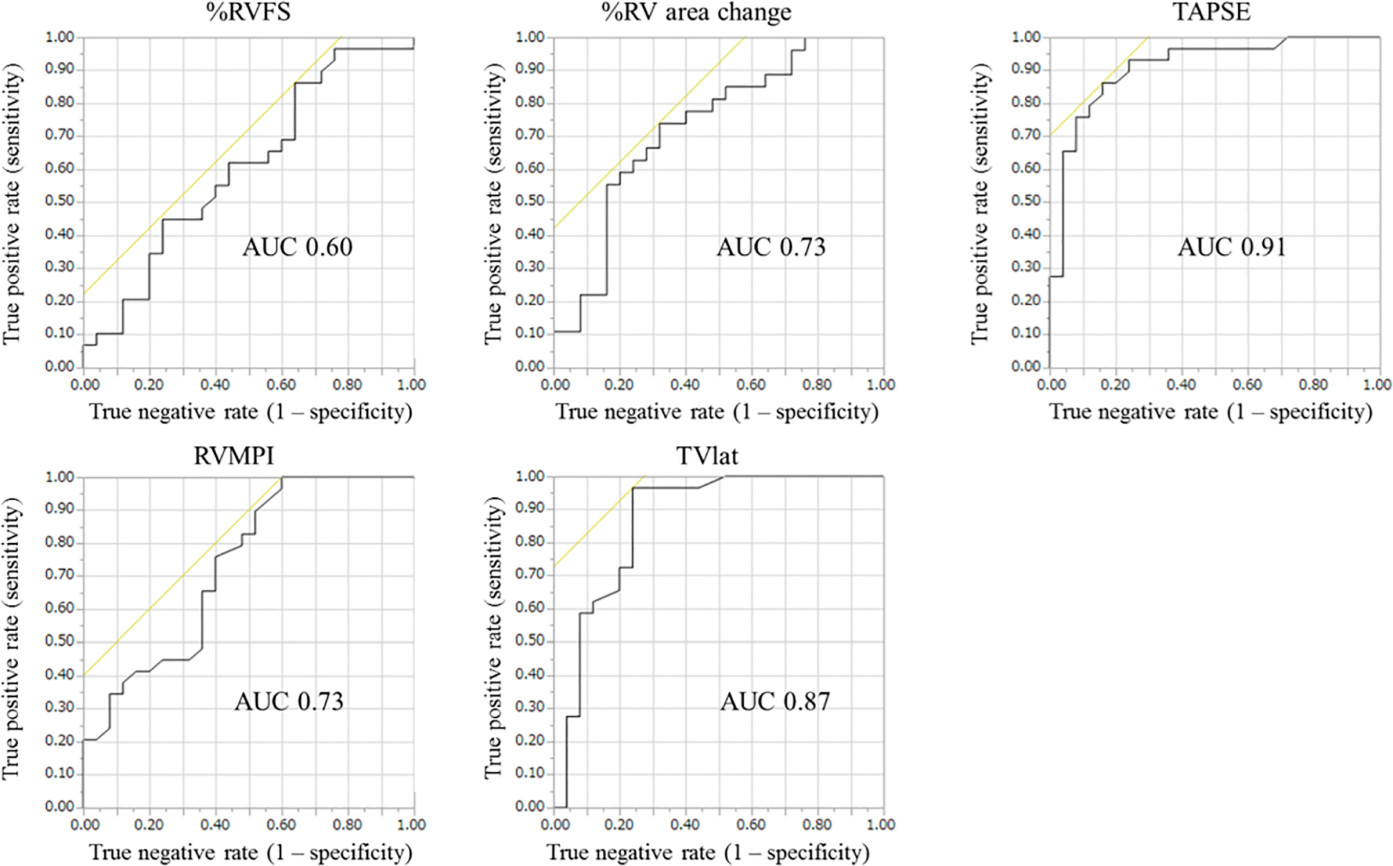
Receiver operating characteristic curve analysis of the accuracy of echocardiographic parameters to detect a significant improvement in CMRI-derived RVEF. %RV area changes at baseline and follow-up were not calculated in two patients (one PAH, one non-PAH) because of poor image quality, whereas the other four echocardiographic indices were evaluated in all 54 patients. CMRI, cardiac magnetic resonance imaging; RV, right ventricular; RVEF, right ventricular ejection fraction; RVFS, right ventricular fractional shortening; TAPSE, tricuspid annular plane systolic excursion; RV MPI, right ventricular myocardial performance index; TVlat, systolic lateral tricuspid annular motion velocity.

The ROC analysis (Fig 3) indicated that the accuracy of the echocardiographic parameters in identifying a prespecified RVEF increase of > 2.9% was low for RVFS (area under curve [AUC], 0.60), moderate for %RV area change (AUC, 0.73) and RV MPI (AUC, 0.73), and high for TAPSE (AUC, 0.91) and TVlat (AUC, 0.87). Similar results were obtained in the subgroup analysis; TAPSE and TVlat had higher AUCs in both groups. In the PAH group, the AUCs were 0.50 for RVFS, 0.62 for %RV area change, 0.83 for TAPSE, 0.55 for RV MPI, and 0.77 for TVlat. In the non-PAH group, the AUCs were 0.70 for RVFS, 0.81 for %RV area change, 0.97 for TAPSE, 0.87 for RV MPI, and 0.96 for TVlat.

### Diagnostic performance of echocardiographic indices to detect improvement in CMRI-derived RVEF

Table 5 shows that the sensitivity, specificity, PPV, and NPV for the detection of CMRI-derived RVEF improvements were higher for TAPSE and TVlat than the other three indices, except for the specificities of RVFS (80%) and %RV area change (84%), which were higher than those of the other three indices. The results of the subgroup analysis were similar to those of the overall analysis, indicating the better sensitivity, PPV, and NPV of TAPSE and TVlat.

**Table 5:** Diagnostic performance of echocardiographic indices to detect significant improvement in CMRI-derived RVEF.

	Sensitivity	Specificity	PPV	NPV
**All PH patients (n = 54)**				
RVFS	33.3	80.0	57.1	60.0
%RV area change*	40.9	80.0	60.0	64.9
TAPSE	83.3	70.0	69.0	84.0
RV MPI	58.3	63.3	56.0	65.5
TVlat	100.0	66.7	70.6	100.0
**PAH patients (n = 23)**				
RVFS	40.0	69.2	50.0	60.0
%RV area change*	44.4	61.5	44.4	61.5
TAPSE	90.0	61.5	64.3	88.9
RV MPI	50.0	46.2	41.7	54.5
TVlat	100.0	61.5	66.7	100.0
**Non-PAH patients (n = 31)**				
RVFS	28.6	88.2	66.7	60.0
%RV area change*	38.5	94.1	83.3	66.7
TAPSE	78.6	76.5	73.3	81.3
RV MPI	64.3	76.5	69.2	72.2
TVlat	100.0	70.6	73.7	100.0

*%RV area changes at baseline and follow-up were not calculated in two patients (one PAH, one non-PAH) because of poor image quality, whereas the other four echocardiographic indices were evaluated in all 54 patients. PPV, positive predictive value; NPV, negative predictive value; RVFS, right ventricular fractional shortening; RV, right ventricular; TAPSE, tricuspid annular plane systolic excursion; RV MPI, right ventricular myocardial performance index; TVlat, systolic lateral tricuspid annular motion velocity

## Discussion

The present study demonstrated significant correlations between the changes in five echocardiographic parameters of RV systolic function and those in CMRI-derived RVEF in precapillary PH patients. Among the five echocardiographic indices, TAPSE exhibited the highest correlation coefficient (r) with the changes in CMRI-derived RVEF. The diagnostic performance of TAPSE and TVlat were higher than that of %RV fractional shortening, %RV area change, and RVMPI in the overall and subgroup analyses. The ability of the echocardiographic indices to identify improvements in CMRI-derived RVEF varied, with TAPSE and TVlat having the highest ability.

Studies have shown a promising clinical role of serial assessment of RV function in PAH.[8, 27, 28] In particular, CMRI studies have shown a prognostic value of baseline and follow-up evaluations of RV wall motion/RVEF in PAH patients.[8, 27] Therefore, the recent guidelines recommended the use of CMRI-derived indices of RV function for monitoring of PH.[27] In contrast, such serial assessments by echocardiography were not recommended by the same guidelines because of limited evidence supporting their clinical relevance. However, recent studies have shown that serially assessed echocardiography indices are associated with clinical outcomes of PH, including survival.[29–32] In the present study, we documented a clinically acceptable accuracy of serially assessed echocardiographic indices of RV function, in addition to their established clinical relevance as baseline measurements.[10, 12] We believe that recent publications, including our present study, provide a rationale for further application of echocardiography in the management of PH.

Among the five echocardiographic parameters, TAPSE and TVlat appeared to provide better accuracy in monitoring of RV systolic function. Such superiority of TAPSE and TVlat has been reported in cross-sectional studies[10] and the present study indicated a similar promising value of the two indices for serial assessment. For example, as shown in Table 4, increases of > 1.1 mm in TAPSE and > 0.25 cm/s in TVlat reflected a significant improvement in RVEF, with sensitivity of 83.3% and 100%, respectively. Alternatively, the sensitivities of RVFS, %RVAC, and RV MPI for detecting improvement in RVEF were low at 33% to 58%. These results may be associated with the methodological features of TAPSE and TVlat measurement; these two parameters reflect longitudinal plane movement, which is more predominant in the right ventricle than in the left ventricle, which contracts predominantly along the short-axis plane.[33]

In a recent report by Spruijt et al., associations between changes in echocardiographic and CMRI indices were examined in 38 PH patients.[19] Similar to the present study, their study exhibited significant associations between CMRI-derived RVEF and four echocardiographic indices of RV function (i.e., TAPSE, %RV area change, fractional longitudinal wall motion, and transverse wall motion). However, the sensitivities of these indices in detecting a decline in CMRI-derived RVEF were low at 33% to 56% and the authors concluded that such parameters were not suitable for serial assessment of RV systolic function in precapillary PH patients. The differences between their study and ours suggest that diagnostic accuracy may vary according to the characteristics of the PH patients or target outcomes. For example, the etiology of PH, treatment regimen and response, and ethnicity are known to affect RV morphology and function.[34–36] Also, in the study by Spruijt et al., sensitivity was calculated to detect a decline in CMRI-derived RVEF, whereas our study calculated sensitivity to detect an improvement in CMRI-derived RVEF. Therefore, the optimal echocardiographic indices of RV function need to be carefully selected depending on the purpose of evaluation and patient characteristics.

The clinical relevance of serial assessment of RV function has been less frequently reported for non-PAH PH patients than PAH patients. Recent studies, however, suggested there is clinical value in serial RV evaluation, particularly in CTEPH patients. Mauritz et al. and others have reported improved RV structure and/or performance in CTEPH patients after interventional procedures, such as pulmonary endarterectomy and balloon pulmonary angioplasty.[37–39] In addition, Kepez et al. reported that improvement in functional capacity of CTEPH patients correlated with improvements in TAPSE or TVlat, rather than with PAP, after pulmonary endarterectomy.[40] Together with these recent publications, the positive results in the present sub-analysis further suggest the potential of echocardiography in the follow-up, treatment optimization, and assessment of non-PAH PH patients.

The reproducibility of the echocardiographic indices should be discussed because the indices are likely to have substantial variation depending on the examiner and/or the condition of the patients. In this regard, we performed a Bland–Altman analysis, calculated the intraclass correlation coefficients, and reported the intraobserver and interobserver variabilities of the five echocardiographic indices in a previous study[10]. In brief, the reproducibility was relatively high for TAPSE and TVlat, whereas it was limited particularly for %RV area change, indicating a patient/examiner-dependent variation in some echocardiographic indices. In addition, compared with CMRI, the image quality of echocardiography can be affected by a patient’s body habitus, bone and/or lung(s), particularly in patients with severe RV dilatation. Indeed, in the present study, %RV area change could not be calculated because of poor image quality in two cases, reflecting the limited reproducibility of echocardiography compared with CMRI.

In the correlation analysis of the changes in the CMRI and echocardiographic indices (Table 4), the correlation efficient (r) of the five echocardiographic parameters ranged from 0.27 to 0.84. From a clinical viewpoint, these values are substantially low because even TAPSE (r = 0.84) explains the 70% variance of the changes in CMRI-derived RVEF, and other indices (r = 0.27–0.72) explain approximately ≤50%. Conversely, ROC analysis revealed acceptable accuracy of TAPSE and TVlat for identifying patients with improved RVEF, with their AUC at approximately ≥0.9. These results suggest that a serial echocardiographic study is better suited for qualitative assessment rather than quantitative estimation of RVEF in precapillary PH.

There were several limitations to this study. First, the present study included only a small number of patients with diverse etiologies of PH, which may have caused inconsistent results. For example, there was a significant association between ΔRVFS and ΔCMRI-RVEF in the overall analysis of the 54 participants, but the association did not reach statistical significance in the subgroup analysis of either the PAH (n = 23) or non-PAH group (n = 31). In addition, regarding the correlation between ΔRVFS and ΔCMRI-RVEF, the weak correlation between the two indices (r = 0.27 in the overall analysis) may have also contributed to the lack of statistical significance in the subgroup analysis. Second, PAH and non-PAH groups were not matched in demographics and pulmonary hemodynamics. This, along with the limited reproducibility/variability of the echocardiographic indices, may have caused inconsistent results between the two groups, i.e., Δ%RV area change correlated with ΔCMRI-derived RVEF in the non-PAH subgroup, but it was not in the PAH subgroup. Third, this was a retrospective observational study with varying reasons and timing of follow-up. Fourth, although some of the PAH-approved drugs may have affected the myocardial function of the patients,[36] the treatment regimens were not controlled. Fifth, the accuracy of echocardiographic indices was examined to detect improvement in RVEF, but this is likely to differ when the target outcome is detection of worsening RVEF. Finally, emerging echocardiographic techniques, such as three-dimensional echocardiography and strain imaging, were not examined in the present study. The promising accuracy and reproducibility of such techniques have been increasingly reported,[41, 42] although their use is still limited because of the need for dedicated equipment and expertise.

## Conclusions

Serially measured echocardiographic indices of RV systolic function significantly correlated with CMRI-derived RVEF. In particular, the changes in TAPSE and TVlat are better correlated with those in CMRI-derived RVEF compared with %RV fractional shortening, %RV area change, and RVMPI in pharmacologically treated precapillary PH patients. The clinical relevance of serial echocardiographic evaluation of RV systolic function of PH patients needs to be further addressed in future adequately-powered prospective studies.
